# Oncology with common sense: beyond biologistic and pharmacological models

**DOI:** 10.3332/ecancer.2026.2059

**Published:** 2026-01-16

**Authors:** Rodrigo Lastra, Patricia Iranzo, Javier-David Benítez-Fuentes, Ana Callejo, Mara Cruellas, Jacobo Gómez Ulla, Isabel Pimentel, José Luis Pérez-Gracia, Marta Ramos, Francisco Gil Moncayo, María Álvarez Alejandro, Marta Gascón, Sergio Martínez Recio, Pilar Rivero, Jorge Bartolomé, Antonio David Lázaro Sánchez, David Fernández Garay, Alicia de Luna Aguilar, Isabel Lorenzo Lorenzo, Joan Brunet, Noemi Reguart, Dario Trapani

**Affiliations:** 1 Rio Working Group, C/Ram de Viu 35 B D, 50006 Zaragoza, Spain; 2 Oncology Department, Hospital Clinico Universitario Lozano Blesa, Calle San Juan Bosco, 15, 50009 Zaragoza, Spain; 3 Oncology Department, Hospital Universitari Vall d’Hebron & Vall d’Hebron Institute of Oncology (VHIO), Passeig de la Vall d’Hebron, 119-129, 08035 Barcelona, Spain; 4 Oncology Department, Hospital General Universitario de Elche, Cami de l’Almazara, 11, 03203 Elche (Alicante), Spain; 5 Oncology Department, Hospital Universitario de Burgos, Calle Ramón y Cajal, 09006 Burgos, Spain; 6 Oncology Department, Hospital Universitario de Badajoz, Carretera de Portugal, 06011 Badajoz, Spain; 7 Oncology Department, Hospital Universidad de Navarra, Av Pio XII, 36, 31008 Pamplona, Spain; 8 Emergency Department, Hospital Clinico Universitario Lozano Blesa, Calle San Juan Bosco, 15, 50009 Zaragoza, Spain; 9 Psycho-Oncology Department (Head), Institut Catalad’Oncologia, Gran via de l’Hospitalet 199-203, 08908 L’Hospitalet de Llobregat (Barcelona), Spain; 10 Oncology Department, Hospital Universitario Miguel Servet, Paseo de Isabel La Catolica, 1-3, 50009 Zaragoza, Spain; 11 Oncology Department, Hospital de la Santa Creu i Sant Pau, Carrer de Sant Antoni Maria Claret, 167, 08025 Barcelona, Spain; 12 Oncology Department, Hospital de Barbastro, Avenida de la Constitucion, 22300 Barbastro (Huesca), Spain; 13 Oncology Department, Hospital Clinico San Carlos & Experimental Therapeutics in Cancer Unit, Instituto de Investigacion Sanitaria San Carlos (IdISSC), Calle del Profesor Martin Lagos, 28040 Madrid, Spain; 14 Oncology Department, Hospital General Universitario Santa Lucia, Calle Santa Lucía, 30202 Cartagena (Murcia), Spain; 15 Oncology Department, Hospital Regional Universitario Málaga, Avenida de Carlos Haya, 29010 Málaga, Spain; 16 Oncology Department, Complejo Hospitalario Universitario de Vigo, Calle Ramon y Cajal, 36202 Vigo (Pontevedra), Spain; 17 Oncology Department, Institut Catala d’Oncologia, Avinguda França, 17007 Girona, Spain; 18 Oncology Department, August Pi Sunyer Biomedical Research Institute (IDIBAPS), Carrer de Villarroel, 170 08036 Barcelona, Spain; 19 European Institute of Oncology, IRCCS, Via Ripamonti, 435, 20141 Milano MI, Italy; 20 Department of Oncology and Hematology-Oncology, University of Milan, Via Festa del Perdono, 7, 20122 Milano MI, Italy

**Keywords:** common sense oncology, patient-centered care, social determinants of health, value-based oncology, clinical and financial toxicity, health inequalities

## Abstract

Rapid advances in oncology have led to unprecedented pharmacological and technological complexity, yet these developments have not always translated into meaningful benefits for patients. Inspired by the Common Sense Oncology movement, this article reflects on the need to recalibrate cancer care beyond biologistic and industry-driven models. We argue for a broader, patient-centred vision of oncology that integrates social determinants of health, prevention, supportive and palliative care, shared decision-making and independent research alongside pharmacological innovation. Drawing on evidence from health economics, clinical research trends and public health, we highlight the paradox between rising oncology expenditures and limited gains in overall survival, quality of life and equity. We also discuss the growing influence of industry-sponsored research, the increasing reliance on surrogate endpoints, and the underfunding of non-pharmacological and psychosocial interventions. Finally, we propose concrete priorities for policymakers, clinicians and research institutions to ensure that oncology progress remains aligned with outcomes that truly matter to patients.

## Times are changing, but the centrality of the patient should not

Almost 2 years ago, a group of oncologists in Spain began meeting to discuss the need for rethinking various aspects of our profession. Our aim was to discuss oncology beyond the industry-driven perspectives and pure pharmacology aspects. Then, we came across the Lancet article published by Common Sense Oncology (CSO) in 2023 [1]. It was a breath of fresh air. We felt that global oncology needed a movement like the one proposed by the CSO. CSO is a global movement that proposes recalibrating cancer care to focus on what truly matters to patients: meaningful survival, quality of life (QoL), equity and informed decision-making. The article points out that many current cancer treatments offer only marginal benefits, while patients face clinical and financial toxicity, as well as loss of time. This inspired our reflection RIO group* to expand this ‘common sense’ approach to the European context and focus, not only on pharmacological aspects, but also primary and secondary prevention, social determinants of health, supportive care, independent research, equity of access and a more humanistic vision of oncology.

Echoing our beloved Nobel laureate Bob Dylan, ‘The times, they are a-changing’, in oncology, this rings especially true. Rapid advances are transforming the field, and especially in the last decade, significant and promising advances have been made in the fight against cancer. When Bob Dylan first began to sing, oncology was only a branch of internal medicine. Now, it has evolved into a highly specialised discipline against cancer, equipped with a vast array of tools and technologies approaching cancer as a battle to be won. In the early 1970s, radiotherapy was one of the first effective treatments for cancer. Then, with the development of early anticancer agents, we became chemotherapeutic doctors. The new century came biological drugs, targeted therapies designed to disrupt specific signals in tumour cells. Over the last ten years, immunotherapy and precision oncology have become important components of treatment algorithms across many tumour types. Multi-omics analysis has generated huge data for drug development and has brought more complexity to the field, focusing on the intrinsic nature of cancer biology mechanisms. However, these advancements have not necessarily aligned with patient needs. Yes, times in oncology ‘are a-changing’, but some things should never change. In this growing medical, pharmacological and technological complexity, are patients truly at the center of these advances? Are they the real protagonists of progress?

## Determinants of health, also in oncology

The World Health Organisation defines health as ‘the state of complete physical, mental and social well-being and not merely the absence of disease or infirmity’ [2]. This definition puts physical, mental and social factors at the same level. In 1974, one of the most revolutionary reports in the field of Public Health, the Lalonde Report, came to light [3]. The Canadian Minister of Health, Marc Lalonde, highlighted the importance of health determinants, arguing that health and disease are influenced not only by biological traits but also by socioeconomic factors, such as income, education, employment, social support and community networks.

How much does each factor weigh in the risk of illness and death? These ranges depend on the source, but it is generally found that human biology contributes approximately 20%–30%, environment 10%–20%, lifestyle 30%–40% and health services 10%–20% to the population’s health status [4, 5]. In 2023, the Spanish Association Against Cancer (AECC), one of the most important patient-led organisation in Spain, launched a campaign addressing all these issues. AECC declared that a person’s postal code has a greater impact than their genetic code on cancer disparities and that a credit card matters more than a health card on cancer outcomes [6]. If this is the case in Spain—a country with a universal healthcare system (UHC)—one can only imagine the weight of social inequalities in oncology worldwide, where UHC for cancer treatments remains far from.

For example, unemployment—a structural issue in Spain—has been associated with higher cancer mortality rates. A review of more than 40 studies investigating the link between unemployment or precarious employment and cancer mortality revealed a clear correlation [7]. Multifactorial causes drive this association. The Lancet published a conclusion during the last economic crisis that remains accurate: ‘*One of the most important health measures to improve the health of populations should be based on guaranteeing decent and stable work, which should be integrated as one of the objectives of public health systems’* [8]*.* According to a Comprehensive Study on Cancer in Europe led by the European Commission and the Organisation for Economic Co-operation and Development over 40% of the cancer burden in Europe can be attributed to preventable risk factors, which are more prevalent among individuals with lower socioeconomic and educational backgrounds. For instance, lung cancer mortality is significantly higher among individuals with lower education levels [9]. The study concluded that ‘*increasing investment in education should also be a priority in the fight against cancer. It is likely the most powerful way to address gender and wealth disparities and to reduce inequalities in health*’ [10].

However, if we consider the investment across different factors, the paradox emerges as the system invests more in those with a lower impact on health outcomes. In Spain, hospital expenditures account for over 60% of health spending, and when pharmaceutical and technological costs are included, this figure rises to nearly 90% [11]. This imbalance persists even in countries like Spain, with UHC and robust healthcare programs.

## Oncology, beyond the ‘mere’ pharmacological aspects

Oncology dominates global healthcare spending, with estimated expenditures on oncology drugs reaching $236 billion in 2024. This represents a doubling or tripling of spending compared to other hospital-based medical areas. Projections indicate a 10% annual increase in the coming years [12]. Nevertheless, has this exponential increase in pharmaceutical spending translated into proportional improvements in patient survival or QoL?

Evidence-based medicine (EBM), with randomised controlled trials (RCTs) as its cornerstone, has represented a significant advance in oncology. By emphasising the pursuit of comprehensive evidence, EBM has often implemented truly effective interventions and discarded practices with little or no value that were previously followed without scientific proof. EBM has helped to identify effective interventions and standardising practices and procedures, beyond individual experience or subjectivity. It has also facilitated a transversal dissemination of knowledge. However, RCTs are time-consuming, heavily regulated and very expensive, leading to a reliance on industry funding. Usually, with an under-representation of certain populations compared to academic non-sponsored trials [13].

A review of 225 RCTs published in highlighted journals found the active role of the pharmaceutical industry in trial design (70%), data analysis (68%) and manuscript writing (66%). Indeed, industry sponsorship was associated with a 3.6-fold higher likelihood of positive conclusions and shorter publication timelines [14]. In the same line, a separate analysis of 694 RCTs revealed that 71% were sponsored by the pharmaceutical industry. These trials were larger, more likely to yield positive results, and were published in higher impact journals as compared with non-industry-sponsored trials [15]. According to a 2021 paper in JAMA Oncology, this trend has increased in the last decades rising from 25% in the late 1980s to 60% by 2004, reaching 85% in 2020 [16]. Moreover, patient’s enrollment in oncology industry-sponsored trials increased from 4.6 to 8.1 between 2008 and 2022, compared to federally funded trials underlying a growing public reliance on industry to conduct clinical research [17]. Additionally, there have been changes in primary endpoints. Overall survival (OS), as a primary endpoint of RCTs, has decreased, while progression free survival (PFS) as a surrogate for OS has increase [16], although PFS has not been shown to be a solid surrogate in most oncology settings [18, 19].

Although industry-supported research has undeniably advanced oncology, other counterbalances are needed. If this trend continues, it can lead to exclusively economic interest with its high cost, its opacity and its clinical and financial toxicity [20]. The psychosocial aspects, collective causes of disease and non-pharmacological interventions, may be relegated due to the difficulty of demonstrating their efficacy through RCTs. The individual causes of the disease and, therefore, the possible solutions (most often pharmacological) can take precedence over the collective causes of the disease and non-pharmacological responses.

## Challenges and opportunities. Focus research and funding that matters to patients

Consider the paradox of drug spending for incurable diseases. The cost of second and subsequent lines of treatment has increased among patients nearing the end of life. There is substantial over-utilisation of marginal, toxic and expensive medicines (especially near the end of life) [21]. In patients with advanced disease who have progressed to a first-line treatment, there are drugs that cost up to 200,000 dollars with an OS benefit of only a few months. Although treatment should still be warranted even at palliative stages if it meaningfully improves QoL, this point can be criticised, as several RCTs prioritise time-based endpoints over patient-reported outcomes or symptom improvement. Problematic trials, dubious endpoints, small effect size, high prices and this is with the consequences of clinical toxicity, financial toxicity [22], time toxicity [23, [24] and efficacy-effectiveness gap [25]. On the other hand, 40% of cancer drugs declared essential by the WHO (excluding immunotherapy and targeted therapy) are only available at full cost to patients in low- and middle-income countries [26].

In Spain, we observe similar paradoxes. We are proud that our healthcare system provides costly treatments to individuals without financial resources. However, other needs—which, in the oncological process, may be of greater concern to the patient (decent housing and caregiver)—are barely funded. With a fraction of the treatment cost, these patients could significantly improve their living conditions. Similarly, expensive drugs are available for elderly patients; an 85-year-old patient who has exhausted three prior treatment lines may receive a new drug costing thousands of euros monthly with minimal restrictions, yet have no access to other core (and more effective) therapeutic supports and interventions. While highly-effective treatments (even if expensive) should be funded and available, it is often the case that these late-lines treatment portend minor improvement in patient outcomes while weighing in toxicity. However, it remains challenging to secure assistance for their basic needs, such as cooking, bathing or grocery shopping, which cost far less—and that address a highly relevant aspect for survival, QoL and people’s dignity. Palliative care and psycho-oncology are also underfunded; Spain’s ratio of palliative care units per capita is among the lowest in the European Union [27].

Society, through its public institutions and as a guarantor of the achievements of our communities, must play a leading role in reshaping oncology. Public administrations need to become much more involved in EBM, and expand the focus on an oncology that matters to patients, beyond technological and pharmacological aspects:

1. Improve care, especially at the end of life, including palliative care and psycho-oncology units.
2. Promote empathetic communication that addresses patient´s inner life and spirituality.
3. Incorporate shared decision-making into care processes.
4. Evaluate in each therapeutic strategy, not only of clinical toxicity, but also of time toxicity and financial toxicity for patients, as well as possible solutions.
5. Prioritise non-pharmacological interventions, such as exercise and nutrition, with preventive and curative potential [28].
6. Empower independent research through public funding for academic and non-profit studies, focusing on neglected areas like rare diseases, comparative studies between different therapeutic options and preventive measures.
7. Foster excellent Academic Translational Research in human biological samples, to identify and validate biomarkers that enable to performance of rational drug development that reduces costs and toxicity of cancer treatments.
8. Encourage the pharmaceutical industry and academia to prioritise patient-centered research goals, such as QoL and OS, while mitigating clinical and financial toxicity. Ensure open data accessibility to support further studies.
9. Promote a change within the regulatory authorities for standardised trial endpoints, inclusion of PROs and safety reporting requirements.
10. Support global public health interventions to address disparities in low-income countries and regions.

* Note: *The Río Group* (https://repositorioindependientedeoncologia.blogspot.com/) *is a space for dialogue and reflection on oncology, grounded in the independence and voluntary commitment of its collaborators (professionals dedicated to cancer care) and the absence of any external funding. Inspired in the Common Sense Oncology movement, it has begun work on the generation, interpretation and dissemination of scientific evidence that places at its center the issues that matter to patients.*

**Figure:**
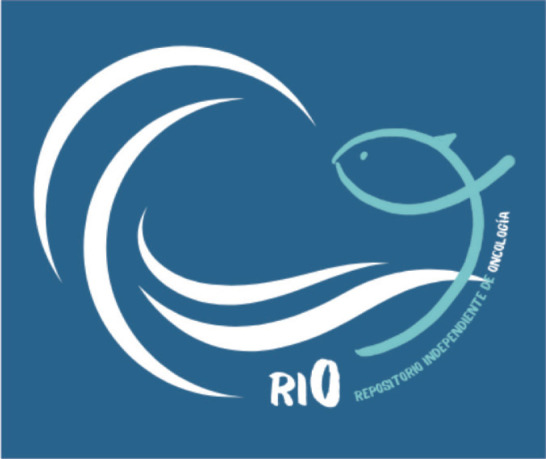


## Conflicts of interest

Rodrigo Lastra reports speaking and travel/congress support from AstraZeneca, BeOne, Eli Lilly and Janssen; Patricia Iranzo reports consulting fees from Boehringer Ingelheim, honoraria from AstraZeneca, Regeneron, Janssen, Pfizer, Takeda and Merck, and travel support from Janssen, Merck and AstraZeneca; Ana Callejo reports honoraria from Bristol Myers Squibb, Roche, Pfizer, Boehringer Ingelheim, MSD and Celgene, and travel/meeting support from Bristol Myers Squibb, Roche, Pfizer, Boehringer Ingelheim, MSD and Eli Lilly; Mara Cruellas reports speaker fees from AstraZeneca and travel support from Pfizer, Eli Lilly, Palex Medical, Novartis, Gilead and Amgen; Isabel Pimentel reports honoraria and/or travel support from the AstraZeneca–MSD Alliance, Gilead, Novartis, Merck and Pfizer, and advisory board participation for the AstraZeneca–MSD Alliance; José Luis Pérez-Gracia reports research grants/support from Amgen, Novartis and Roche, speakers’ bureau/advisory board participation for Amgen, Astellas, MSD, Novartis, and Roche and travel support from Astellas, Bristol Myers Squibb, MSD, Roche and Merck; María Álvarez Alejandro reports speaking and travel/congress support from Sanofi, Roche, Bristol Myers Squibb, Novartis, Amgen, Servier, Merck, Celgene, AstraZeneca and BeOne; Marta Gascón reports speaking and travel/congress support from Roche, AstraZeneca and MSD; Sergio Martínez Recio reports honoraria from Pfizer, AstraZeneca, Pierre Fabre, Sanofi, Bristol Myers Squibb, Takeda, Novartis, Eli Lilly, and Regeneron and travel/meeting support from Roche, Eli Lilly, Merck, Pfizer, Bristol Myers Squibb, Novartis, MSD, Pierre Fabre and Janssen; Pilar Rivero reports speaking and travel/congress support from Roche, AstraZeneca and Eli Lilly; David Fernández Garay reports honoraria from GSK, AstraZeneca, Eisai, Roche, MSD, PharmaMar, Leo Pharma, Novartis, Pfizer and Sanofi, and support for attending symposia from Roche, Novartis, Eli Lilly, Pfizer, Sanofi, GSK, AstraZeneca, MSD and Leo Pharma; Noemi Reguart reports advisory board/committee participation and invited speaker roles for AstraZeneca, Bristol Myers Squibb, Guardant Health, Janssen, MSD, Novartis, Pfizer, Roche, Sanofi and Takeda.

Javier-David Benítez-Fuentes, Jacobo Gómez Ulla, Marta Ramos, Francisco Gil Moncayo, Jorge Bartolomé, Antonio David Lázaro Sánchez, Alicia de Luna Aguilar, Isabel Lorenzo Lorenzo, Joan Brunet and Dario Trapani declare that they have no conflicts of interest.

## Funding

This study received no specific funding from public, commercial, or not-for-profit funding agencies.
